# Hypnosis and superficial cervical anesthesia for total thyroidectomy in a high-risk patient – A case report

**DOI:** 10.1016/j.ijscr.2020.05.078

**Published:** 2020-06-06

**Authors:** P. Makovac, A. Potié, A. Roukain, L. Pucci, T. Rutz, P.A. Kopp, M. Matter

**Affiliations:** a Department of Visceral Surgery, University Hospital of Lausanne and University of Lausanne Switzerland; b Department of Anesthesiology, University Hospital of Lausanne and University of Lausanne Switzerland; c Division of Endocrinology, Diabetology and Metabolism, University Hospital of Lausanne and University of Lausanne Switzerland; d Service of Cardiology, University Hospital of Lausanne and University of Lausanne Switzerland

**Keywords:** Thyroidectomy, Superficial anesthesia, Hypnosis

## Abstract

**Introduction:**

Total thyroidectomy can be challenging in high-risk patients. Local cervical anesthesia with sedation is an alternative to general anesthesia.

**Case presentation:**

A 33-year old male patient with cyanotic congenital heart disease due to unrepaired tricuspid atresia type Ic and associated pulmonary arterial hypertension presented with tachycardic atrial fibrillation and amiodarone-induced thyrotoxicosis resulting in recurrent hemodynamic instability. Because of difficulties controlling the thyrotoxic state, the indication for total thyroidectomy was established. Total thyroidectomy was subsequently performed using local anesthesia combined using a hypnosis-analgesia technique instead of intravenous sedation. The intervention and the post-operative course were uneventful.

**Discussion:**

A well-established therapist-patient relationship is crucial for a successful induction of hypnosis. Patient motivation and expectations are equally important for a successful implementation of this approach.

**Conclusion:**

We conclude that hypnosis combined with local anesthesia provides an effective alternative in selected patients with very high anesthesiological risk.

## Highlights

• Total thyroidectomy can be challenging in high-risk patients.
• Local superficial anesthesia combined with a hypnosis-analgesia technique instead of intravenous sedation.
• Because of difficulties controlling the thyrotoxic state.
• Given the multiple cardiac and large vessel malformations, a possible variant of the inferior laryngeal nerve was expected.
• Locoregional deep cervical anesthesia can be associated with breathing problems when performed bilaterally.

## 1 Introduction

Total thyroidectomy under loco-regional cervical anesthesia associated with sedation is a well-known technique that has been used for more than a century [1]. More widely used in the past, it is now usually restricted to patients with contraindications to general anesthesia [2]. In a high-risk patient, we opted to perform total thyroidectomy with superficial cervical anesthesia under hypnosis rather than with sedation with benzodiazepines or other sedative drugs. This work has been reported in line with the SCARE criteria [3].

## 2 Case report

### 2.1 Clinical presentation

**Fig. 1:**
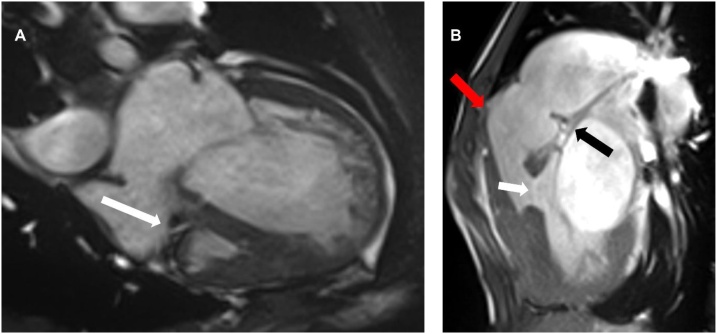
Cardiac magnetic resonance imaging. A) Four-chamber view with visualization of the atretric tricuspid valve (arrow) and the residual right ventricle. B) Sagittal view with visualization of the non-restrictive ventricular septal defect (white arrow), the pulmonary valve (red arrow), and the residual non-restrictive pulmonary banding (black arrow).

We report on a 33 year-old male patient followed at our center for a unrepaired tricuspid atresia type IC with unrestricted ventricular septal defect with left-right shunting, a non-restricted atrial septal defect with right-left shunting, and a functional single left ventricle with normal systolic function (Fig. 1). His past medical history included two attempts of pulmonary artery banding at the age of 15 and 19 years at initial presentation at our center, requiring subsequent debanding due to ventricular arrhythmia. The patient declined any further surgical interventions and developed pulmonary arterial hypertension (PAH) class I.4.4. according to the Nice classification [4], with a pulmonary resistance of 7.5 WU and a persisting significant left to right shunt (Qp:Qs = 2.5 :1). Oxygen saturation at room air was 86 %. The patient is also known for cardiac cachexia, asthma without allergic components, and a restrictive syndrome of extra-pulmonary origin (mainly due to scoliosis and post-thoracotomy status). He developed secondary erythrocytosis and suffered a single episode of vertebrobasilar transient ischemic attack (TIA) in 2010, presumably of embolic origin. Anticoagulation with acenocoumarol was started. In 2016, he experienced a first episode of paroxysmal tachycardic atrial fibrillation (AFib) requiring emergency electric cardioversion. At that time, therapy with amiodarone and metoprolol was initiated and anticoagulation changed to apixaban 2.5 mg bid. In 2018, a second episode of tachycardic Afib associated with hemodynamic instability required an urgent electrical cardioversion. While he was euthyroid in the past, thyroid function tests performed in fall 2019 documented overt thyrotoxicosis with a TSH of < 0.005 mUI/l (0.27–4.3), a FT4 of 64 pmol/l (9–19), and a free T3 of 10.4 pmol/l (2.6–5.7) (Abbbott Architect immunometric assays). Antibodies against the TSH receptor were not elevated (0.51 U/l; reference <1.75) A thyroid ultrasound showed a goiter without nodules and with diminished vascularity. The diagnosis of amiodarone-induced thyrotoxicosis (AIT), most likely of mixed nature, was established and treatment with carbimazole and prednisone initiated. The biochemical control remained unsatisfactory despite therapy with carbimazole 40 mg qd, prednisone 40 mg qd, and sodium perchlorate 900 mg qd. In parallel, the patient had recurrent episodes of tachycardic Afib leading finally to a prolonged hospitalization and a further electric cardioversion. The patient eventually developed persistent Afib which remained tachycardic and symptomatic despite the intensification of antiarrhythmic treatment with amiodarone, nadolol, digoxin and diltiazem.

Due to the refractoriness of Afib to medical treatment and the evidence of a newly compromised ventricular systolic function, the indication for semi-elective total thyroidectomy was established. Because of the very high anesthesiological risk due to the compromised cardiovascular and pulmonary situation, the indication for a thyroidectomy under local anesthesia was established in a multidisciplinary meeting. Rather than opting for intravenous sedation, it was decided to use hypnosis. All procedural steps were discussed and explained in detail with the patient. In particular, the anaesthesiologist-hypnotherapist provided thorough information about the hypnosis and local anesthesia. This also included a comprehensive collection of personal data of the patient with a focus on memory, observational capacity, understanding, personal activities, environment, and sensorial aspects [2]. The patient provided informed consent and was willing to accept the risks of a general anesthesia in case of conversion to general anesthesia would have been necessary.

### 2.2 Anesthesia

**Fig. 2:**
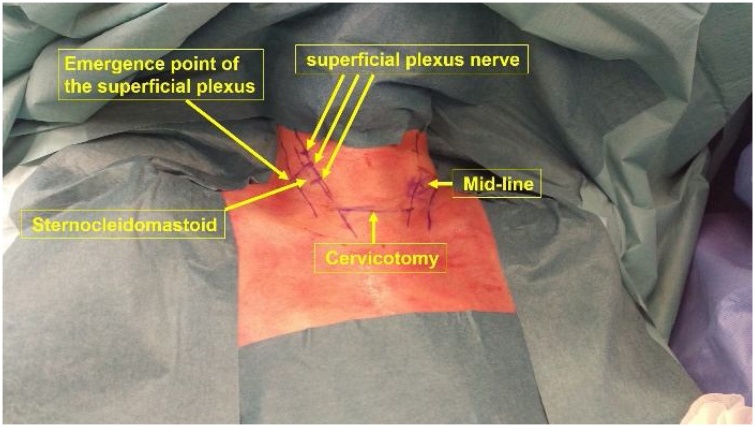
Injection sites for the modified superficial cervical plexus block. A line connecting the mastoid process to the clavicle on the anterior and posterior edge of the sternocleidomastoid was drawn. A total of 29 mL of lidocaine 1% with adrenalin was distributed on both sides as follow: middle of the posterior margin of the sternocleidomastoid (corresponding to the emergence of the superficial cervical plexus), anteriorly and longitudinally at 3 points (cervical branches), in the sternocleidomastoid muscle itself, and along the cervical incision.

After standard monitoring in use in our department, the induction of hypnosis was performed in the operating room. Remifentanil (0.04 mcg/kg/min) was started concomitantly and used throughout the surgery. In order to induce relaxation and calmly modulate perceptions, sensations, and emotions of the patient, permissive suggestions were used by the anaesthesiologist-hypnotherapist. The operating room was prepared accordingly: the anaesthetist's monitoring devices as well as the surgical instruments were silenced, lights were dimmed, staff communicated in soft voice, and access to the room was strictly limited. The patient was placed in a semi-seated position, with his neck in slight hyper-extension. Once the anesthesiologist in charge of hypnosis gave the “go ahead”, the neck was prepared for surgery. As described elsewhere [5], anaesthesia was applied around the sternocleidomastoid muscle. A line connecting the mastoid process to the clavicle on the anterior and posterior edge of the sternocleidomastoid was drawn (Fig. 2). A total of 29 mL of lidocaine 1% with adrenalin was injected on both sides as follows: middle of the posterior margin of the sternocleidomastoid (corresponding to the emergence of the superficial cervical plexus), anteriorly and longitudinally at 3 points (cervical branches), in the muscle itself, and along the cervical incision.

### 2.3 Surgery

Given the multiple cardiac and large vessel malformations, a possible variant of the inferior laryngeal nerve was expected. Intra-operative neuromonitoring was not used. During surgery, a non-recurrent laryngeal nerve on the right side could indeed be identified. Surgery was performed with a harmonic scalpel (Harmonic FOCUS Curved Shear, Ethicon Endo-Surgery) and electro-bipolar forceps. All parathyroid glands were identified and preserved.

Communication with the patient was maintained at all times during hypnosis. The patient was then gradually guided back to full consciousness through controlled, focused breathing. Total hypnosis time was 165 min and surgery lasted 100 min.

In the recovery room, the patient was completely awakened. The intra- and post-operative pain control were evaluated using a numerical rating scale (0–10). The patient recalled almost no pain during the intervention (1/10), but experienced severe sore throat (8/10) during the first post-operative night and the following day, which improved within 48 h (3/10). During the first night in the intensive care unit, no rhythm disturbances requiring treatment were recorded. He developed transient asymptomatic hypocalcemia (2.0 mmol/L), corrected by oral calcium. The post-operative parathyroid hormone level was normal (35 ng/L).

In the immediate post-operative period, the patient complained of extreme fatigue which is a well described consequences of hypnosis [6].

The anatomopathological examination revealed a diffuse goiter (5 × 3.5 × 2.5 on the left side and 6.5 × 3.5 × 2.5 on the right).

Because of recurrent Afib, sinus rhythm was restored by a repeat electric cardioversion. The antiarrhythmic therapy with amiodarone, nadolol and diltiazem was continued. At 4 month follow-up, the interrogation of the loop recorder showed maintenance of sinus rhythm and only short runs of Afib.

## 3 Discussion

The patient reported here illustrates the feasibility of total thyroidectomy with modified superficial local anesthesia in association with hypnosis in a situation with particularly high anesthesiological risk.

Although widely used in the past, there is no routine indication for local anaesthesia due to the safety of modern general anesthesia [7]. Thyroidectomy performed with local superficial cervical anesthesia requires concomitant sedation, and locoregional deep cervical anesthesia can be associated with breathing problems when performed bilaterally. Hypnosis has been used for neck surgery, but this modality is not commonly used [8].

For hypnosis, a well-established therapist-patient relationship is crucial for a successful induction of the hypnotic state during surgery. During a hypnosis session, there are three classical phases guided by the therapist: induction, therapeutic suggestions, and emergence from the hypnotic state [6]. The aim of the first phase is to relax the patient while helping him/her to imagine a quiet place and focus on a pleasant daydream. During the second phase, the therapist provides suggestions to the patient. These are intended to treat, reduce, circumvent potential complaints, symptoms or disorders. In the context of surgery, suggestions for analgesia are commonly provided. During the entire procedure, the collaboration of the patient is necessary. Thus, the hypnotic experience is an active process, and it is not effortless. To improve the patient's receptivity, the therapist uses non-authoritarian hypnosis techniques and guides him or her through the different stages of the surgery. The patient appears to be asleep but neuroimages show a functional activity that is distinct from sleep. The third phase allows the patient to return to a normal state of consciousness.

We conclude that hypnosis, which is dependent on available expertise and patient collaboration, can be a valuable alternative to intravenous sedation in selected high-risk patients undergoing thyroidectomy under local anesthesia.

## Declaration of Competing Interest

There are no conflicts of interest or personal relationships that could have influence the work reported in this paper

## Funding

We received no founding and we had no sponsors.

## Ethical approval

This case report is exempt from ethical approval in our institution.

## Consent

We obtained a clear end well informed consent from the patients to write this case report. and publish the images in anonymous form. Any identifying detail that was not essential was omitted. We did not altered any important patients’ characteristic.

## Author contribution

**Makovac Petra** (corresponding author): writing original draft, conceptualisation, visualisation, editing, investigation.

**Potié Arnaud:** conceptualisation, visualisation, writing, review and editing

**Roukain Abdallah**: writing, review

**Pucci Lorenzo**: writing, investigation

**Rutz Tobias:** writing, reviewing

**Kopp Peter:** conceptualisation, writing, reviewing, editing

**Matter Maurice:** Conceptualisation, writing original draft, reviewing, editing

## Registration of research studies

1 Name of the registry: **This is not a research study, thus it is not registered.**
2 Unique identifying number or registration ID: There is no number
3 Hyperlink to your specific registration (must be publicly accessible and will be checked):
4 There is no link

## Guarantor

Makovac Petra

## Provenance and peer review

Not commissioned, externally peer-reviewed
